# The Subtle Crisis: Public Domain Genomes and the Ethics of Translational Infrastructure

**DOI:** 10.1002/eahr.70032

**Published:** 2026-09-04

**Authors:** Jonathan Edward LoTempio

**Affiliations:** ^1^ Assistant professor of pediatrics and biological chemistry and an associate director of translational bioethics at the School of Medicine at the University of California, Irvine

**Keywords:** human genomic resources, public domain genomes, translational science, translational bioethics

## Abstract

Public domain human genomic resources are infrastructure: tools researchers use to ask basic biological questions whose answers are then translated into products and care. Translational science now asks them to support an expanding set of tasks, including clinical variant interpretation for diverse populations, pharmacogenomic prescribing, polygenic risk prediction, and the training of clinical artificial intelligence. The corpus of public domain genomes, due in large part to upstream recruitment choices, is not fit for these purposes, and the gap between discovery and translation is widening. This essay argues that closing the gap requires treating public domain genomic infrastructure as a particular object of translational bioethics rather than a technical precondition for it. The limited number of genomes in the public domain relative to the broader genomic record, and the typology‐friendliness of how that record represents human variation, are two faces of the same set of upstream choices. Reversing them is not a matter of more sampling under existing terms; it is a matter of building infrastructure of a particular kind; infrastructure made from people. That category, common in genomics but absent from the rest of science, demands an ethical apparatus the field has not yet built. Here we consider the commitments such an apparatus requires, and argue that where, how, and with whom we build genomic infrastructure is itself an ethics question the field has largely declined to ask.

By any reasonable count, fewer than 5,000 whole human genomes sit in the public domain. The figure is approximate. It depends on how one counts Personal Genome Project chapters with inactive data links and how one treats Human Genome Diversity Project samples with variable controversy, and whether one includes reference assemblies whose sequence traces largely to a single individual but includes multiple people.^1^, ^2^, ^3^ The order of magnitude, however, is stable, and small. The most used data come from the 1000 Genomes Project including around 3,200 participants and the Genome in a Bottle consortium hosted by the US National Institute of Standards and Technology (NIST), which includes seven participants. Against the tens of millions of genomes already sequenced worldwide across research cohorts, national biobanks, and clinical services, the public domain fraction is close to a rounding error.

The asymmetry would be tolerable if one believed public domain genomes were a finished achievement, a set of reference resources laid down by earlier, wiser reference building projects and now serving as adequate scaffolding for whatever comes next. This essay argues that the belief is wrong, that it is increasingly costly, and that translational bioethics, as Rothstein and others have defined it in this column and elsewhere,^4^, ^5^ is the right subdomain in which to consider the impact.

It is worth saying directly what kind of thing public domain genomic resources are. They are not medical studies that happen to release their data. They are research infrastructure, of the same general category as the standard reference materials curated at NIST and other standards agencies or telescopes and particle accelerators around the world. Like those tools, they exist to enable other research; their value is in what others do with them, downstream and over decades. Cribb argued more than a decade ago that translational ethics is the project of bridging the theory‐practice gap in medical ethics,^6^ and Rothstein's further work has shown that the bridge runs through the social and institutional implications of new translational science.^5^

Public domain reference projects sit on that bridge, and they sit there uneasily. They are infrastructure, but they are infrastructure made from participant people. Their constituent matter is not glass and metal; it is those contributors who retain some interest in their contribution after they have made it, and who must consent to participate in a research legal regime, not procured by offices for construction. No other class of major science infrastructure is built this way. That asymmetry is the ethical fact this essay takes seriously, and it is what makes public domain genomic infrastructure a particular case translational bioethics has the focus, but not yet the apparatus, to address.

Recently, it has been argued that the very concept of broad data sharing in genomics has been used imprecisely and interchangeably.^7^ That imprecision obscures how narrow the genuinely public domain portion of broadly shared data actually is relative to the total number of genomes produced. The existing public domain corpus was largely designed in the pursuit of answering basic science questions. It is not fit for the new tasks translation now asks of it: variant interpretation, pharmacogenomic prescribing, polygenic risk prediction, and the training and validation of clinical artificial intelligence, each of which depends on representing populations that are themselves continuously varying and unstable. The specific inadequacy of the public domain corpus is that, due to its small size, its structure under analysis tracks recruitment location more than biological reality.

What basic and translational science builds downstream of public domain infrastructure is only as good as the reference data supporting it, and a public domain so limited by recruitment location relative to the total human population produces uneven results. The adequacy of the substrate is therefore not a technical question that bioethics inherits from genomics; it is a question the field is responsible for advocating to rectify.

## WHAT THE PUBLIC DOMAIN HOLDS

Human genomic reference data sit in an unusual position. Some data are public in the strongest sense: downloadable and released under terms that permit unrestricted use. Other data are controlled‐access, available only through data‐use agreements that restrict some aspects of their reuse. Still other data

**Genomics has a bad track record of including enough people from varied backgrounds, and data protection is an important aspect of the rights people hold.**

are proprietary, held by biobanks and sequencing services whose participants consented to research use but not to release. And a growing class of data lives in semipublic limbo: deposited in repositories with broken links, migrated to platforms that have since changed access terms, or governed by consortia whose active custodians have moved on.

The resources most researchers would name as genuinely public domain are familiar. The current human reference assembly traces primarily to a single anonymous donor, annotated as RP‐11. The 1000 Genomes Project's high‐coverage release includes approximately 3,200 participants drawn from 26 populations across five continents. The Personal Genome Project's US and UK chapters include roughly 1,100 participants who consented to fully open release. NIST Genome in a Bottle is built around seven individuals with carefully characterized variation. The Human Pangenome Reference Consortium has begun adding depth: its first draft release in 2023 provided 47 phased diploid assemblies from a cohort of genetically diverse individuals drawn from the 1000 Genomes Project, with plans to expand to newly recruited individuals.^8^

Two features of this inventory matter. First, these resources were not built to a common specification. The reference genome was built to be a substrate for alignment of lower‐quality genomes, not a population sample; 1000 Genomes was built to characterize common variation; Genome in a Bottle was established as a validation tool; the Personal Genome Project was built as a demonstration of open consent. None was designed to provide adequate reference data for translational science. Second, as argued elsewhere, many of the decisions made during the Human Genome Project, in a 1990s policy environment that differed substantially from the present one, continue to shape what reference data we now possess and what we can and cannot do with them.^9^ The consent instruments, anonymization practices, and institutional arrangements of that era persist in today's resources, and they were not designed with contemporary translational uses in view.

## WHY TRANSLATION CANNOT MAKE DO

The gap between basic‐science use of public domain resources and the application of these findings and tools to translational science, the discipline National Institutes of Health (NIH) has organized around the National Center for Advancing Translational Sciences since 2011,^10^ is not abstract. Three concrete areas show it clearly.

### Variant interpretation in rare and mendelian disease discovery

When a clinician orders a diagnostic panel, each returned variant must be classified along a spectrum from benign to pathogenic under joint guidelines of the American College of Medical Genetics and Genomics and the Association for Molecular Pathology.^11^ Classification depends on how often a variant has been observed in ostensibly healthy populations. Population‐frequency evidence is only as good as the populations sampled; variants common in groups missing from reference data may be misclassified as rare and, by extension, potentially pathogenic. This rears its head in rare disease research, where the clinical pipelines can fail. One deeper problem is that human migration and social change make populations themselves unstable. In the absence of reference data that capture the full continuum of human variation, individuals are compared to whatever named groups happen to be nearest to them in the available data, and that comparison may be a poor match for their actual ancestry.

### Polygenic risk scores

Polygenic models trained in one ancestry typically lose substantial predictive accuracy when applied to another. Martin and colleagues showed that current polygenic scores are several‐fold more accurate in “European‐ancestry” individuals than in others, a consequence of Eurocentric biases in the genome‐wide association studies on which the scores are trained.^12^ The problem has intensified as polygenic scoring has moved from research into commercial disease‐prevention products. While polygenic‐score development is less constrained by the smallness of the public domain than other tasks (the relevant association studies use far larger cohorts), the disparities reveal that even with abundant data, the same upstream choices about how human variation is partitioned into named groups carry forward, and so does the uneven performance that follows.

### Artificial intelligence

Foundational models in genomic medicine are trained on whatever data their developers can assemble. Public domain data, being public, disproportionately shape models released openly, benchmarked competitively, and audited publicly. Private data disproportionately shape models whose training‐set composition cannot be inspected from outside the firm that built them. When the public domain represents a small share of the data on which models can be trained, model development falls to whoever holds the larger private share, and auditing falls to no one.

In each domain, the missing data are not randomly missing. Popejoy and Fullerton showed nearly a decade ago that individuals of non‐European descent remained starkly underrepresented in genome‐wide association studies, and that such gains as had been made were concentrated in Asian cohorts rather than evenly distributed.^13^ The structural shape of that gap tracks existing health inequities, and it will reproduce those inequities downstream unless it is closed.

## A TENSION OF TYPOLOGY

The 2023 report by the National Academies of Sciences, Engineering, and Medicine (NASEM), *Using Population Descriptors in Genetics and Genomics Research*, arrived as a direct challenge to how researchers group participants. Its first recommendations are the most important: researchers should not use race as a proxy for genetic variation; researchers should avoid typological thinking that presumes hierarchy, homogeneity, or temporal stability of groups; researchers should attend to the connotations of the labels they use.^14^

**Figure 1:**
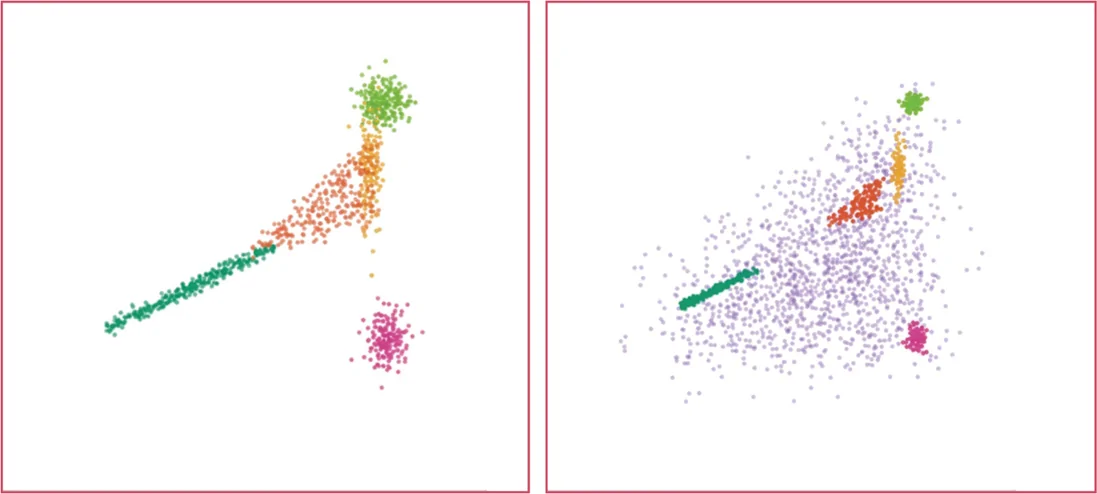
The Public Domain Corpus Produces Apparent Groups That Broader Sampling Could Dissolve Schematic scatterplot rendered to appear like genomic principle components analysis (PCA) scatter plots. The left panel approximates the cluster pattern visible in widely used public domain reference data such as the 1000 Genomes Project, in which a small number of recruited populations resolve into visually tidy groups separated by largely empty space. The right panel shows the same anchor regions at reduced size, surrounded by a haze representing the continuous human variation that denser, less recruitment‐driven sampling could surface. Neither panel depicts real participants. The figure is intended to show that the appearance of discrete groups in the existing public domain is an artifact of sampling strategy rather than a reflection of clear biological boundaries between present‐day populations, and that what looks empty in the left panel is unsampled rather than absent.

These recommendations sit in tension with how reference data are routinely presented. Principal components analysis (PCA), a standard first look at a new cohort, produces visual clusters. Clusters invite grouping; grouping invites names; names invite the very typological thinking that NASEM warned against. A reference corpus small enough to produce visually tidy clusters is one that makes typological thinking easy to sustain. A larger, more variegated corpus, in which the space between named groups is well‐populated rather than blank, makes typology harder to maintain and the continuity of human variation more obvious (see figure 1). Corpus composition is therefore not ethically neutral with respect to NASEM's framework. It actively supports or undermines the mode of thinking the framework seeks to displace.

This is the first place where translational bioethics can offer something discovery‐era bioethics cannot: treating the size and variation of data in the public domain as a particular object of ethical attention rather than general concern over participation in genomics.

## TRANSLATIONAL BIOETHICS‐INFORMED FUTURES FOR MORE CONTINUOUS REFERENCE RESOURCES

Genomics has a bad track record of including enough people from varied backgrounds, and data protection is an important aspect of the rights people hold. A translation‐ready public domain infrastructure that represents the continuity of human diversity is not, however, going to come from doing more of what produced the clustered dataset. Focused sampling from pre‐named populations does not just produce a small corpus; it produces a corpus organized around the categories used to recruit it, which is why the existing public domain looks tidy under PCA in exactly the way that NASEM warned against. Building a public domain resource that supports translation requires different choices, not larger versions of the same ones, and those choices are bioethics decisions with technical consequences rather than technical decisions with bioethical implications.

### Purpose‐specific consent

Genomics‐era broad consent has been used to enable an indefinite set of downstream uses: variant‐classification queries, pharmacogenomic lookups, polygenic‐score training, AI model training, and uses not yet invented. Rothstein, Rajasekaran, and Dove have argued in this column that the open‐ended broad consent currently used by biobanks for health records is an under‐addressed privacy problem and have called for time limits and periodic reconsenting.^15^ Reference projects whose purpose is public domain release are a special case, because the contribution is by design irreversible and the participant relationship effectively ends at enrollment. That is precisely why purpose‐specific consent matters here: the conversation at recruitment can be candid about what public domain release entails for that particular contribution, rather than asking participants to authorize an open‐ended set of future uses they cannot foresee.

### Public and private resources for public benefit

The case for public domain release is clearer now than at any time in the past two decades, in part because its absence is becoming visible. When 23andMe entered bankruptcy in 2025, it was argued that the proposed sale of participant‐derived data threatened the public benefit of scientific contributions built on participant goodwill, and that such data should not be treated as ordinary commercial assets available to the highest bidder in an insolvency proceeding.^16^ That particular case resolved with a nonprofit acquisition: TTAM Research Institute, founded by 23andMe cofounder Anne Wojcicki, completed the purchase of 23andMe's assets in July 2025 under Section 363 of the US Bankruptcy Code.^17^ The acquisition prevented enclosure for now, but it did so through a sale process and a structure that depended on a particular bidder being willing and able to outbid another commercial buyer. There is no general guarantee that data at this and other private entities will remain available to researchers under fair, reasonable, and nondiscriminatory terms. A public domain alternative is the structural insurance against the next iteration of this problem.

### Community‐embedded capacity

Doerr and Yu have argued in this column that big‐health‐data research now operates at the level of communities rather than individuals, and that the harms it risks are correspondingly collective.^18^ Projects to increase the number of public domain genomes can support that argument rather than threaten it. The answer is not to refuse such projects; it is to build them inside and with the communities whose data they hope to represent. Community‐embedded capacity means local investigators with decision rights, local ethics review that is not pro forma, and local capacity to use and interpret the data generated.

### Distributed infrastructure

The capacity to store, curate, and maintain genomic reference data is not globally distributed. Concentrating stewardship in a small set of jurisdictions concentrates governance authority alongside it. Andanda, Machinya, and Mutomba's recent essay here makes the corresponding argument for enhanced data governance in Sub‐Saharan Africa.^19^ Combined with community‐embedded capacity building, that argument extends naturally to the physical and institutional layer beneath the data, where it applies with at least equal force.

### Public‐values legitimacy

Evans has argued that translational science is justified by appeal to public interest but has no mechanism for determining what the public actually wants.^20^, ^21^ Infrastructure whose entire rationale is that it serves the public is the obvious place to take that argument seriously. It is also where doing so is hardest, because infrastructure decisions are made in many places, over long time scales, by people whose incentives are organizational rather than public. Rothstein and colleagues recently surveyed research‐ethics review across 22 countries and found that, of those countries, the United States is the only one whose institutional review boards (IRBs) are explicitly prohibited from considering the long‐term societal implications of biomedical research.^22^ That regulatory asymmetry sits awkwardly against any infrastructure project whose ethical case rests on those very implications.

## PARTICIPATING IN PUBLIC DOMAIN SHARING

The arguments above all converge on a single uncomfortable question: whether it is ethical to ask participants to release their genomes into the public domain at all. A skeptic might say that public domain release is a uniquely heavy “ask,” particularly vulnerable to coercion and regret, and incompatible with the participant‐centered direction in which research ethics has moved.^22^ The skeptic's deeper objection is that public domain release misclassifies what genomic participation is by trying to apply an open‐science model to what is, fundamentally, a medical study. Medical studies do not as a matter of routine practice get released to the world.

The reframe in the introduction is the answer. Public domain reference projects are not medical studies that happen to release their data; they are infrastructure projects, and the contribution participants make to them is closer to the contribution one makes to a public good than to participation in a clinical trial. The asymmetry of public domain genomic infrastructure within the broader infrastructure category is that it is built from people, and the people retain interests in their contribution. The “ask” is therefore real and heavy, but it sits in a different ethical category than the medical study ask. The medical study consent apparatus does not quite fit, because it was developed for a different kind of relationship. The honest enrollment conversation is then not a workaround for an ethically dubious ask; it is the appropriate form of the relationship for that category of contribution.

A recent paper has argued that, given the changing meaning of “broad” in data sharing, the field should consider whether participants ought to be encouraged to share their status as research participants publicly.^7^ That question pushes on a deeper one genomics has rarely tested. Public domain release is qualitatively different from ordinary research participation, and it is worth asking whether the smaller group of participants who agreed to it understood, at the time of agreement, the scope of what they were agreeing to.

Most consent forms in genomics are written to manage institutional liability and to satisfy IRBs. Few are written to have the conversation a person needs to have before agreeing that their genome will be downloadable by anyone, anywhere, for any purpose. Public domain release is the strongest commitment a participant can make to an open scientific commons, and a translation‐era version of that commitment has to acknowledge risks the discovery‐era version did not. Top of mind should be that there may be:

- Re‐identification under future technical conditions the participant cannot anticipate.
- Use by commercial actors the participant did not endorse, with inferences about relatives who never consented.
- Inferences about descendants who do not yet exist.
- Uses to train clinical tools that will be deployed on populations the participant belongs to.

Some of these risks were imaginable in the 1990s and some were not, and the difference is exactly what a translation‐era ask of participants has to address. The structural conditions of public domain reference projects compound this problem. There is no ongoing study from which to withdraw, no recontact pathway through which terms can be renegotiated, and once data enter the public domain, no institutional actor retains effective authority to recall them.

The 2024 revision of the Declaration of Helsinki reframed research governance around meaningful engagement with participants before, during, and after studies, replacing “research subjects” with “research participants” to signal the shift from objects of inquiry to active agents.^23^ Rothstein has further argued in this journal for a research‐participant bill of rights that places the burden of meaningful disclosure on the asker rather than on the form.^24^ For low‐touch reference projects, the during‐and‐after engagement Helsinki demands cannot be supplied; engagement must happen before and at enrollment, or it cannot happen at all.

The standard framing treats fuller disclosure as a burden on recruitment: the more honestly risks are named, the lower enrollment will be. That framing assumes participants are rational only in a narrow sense, weighing self‐interested risk against modest individual benefit, and it assumes the conversation at enrollment is a hurdle to clear rather than the place where governance lives. The evidence from community‐engaged research suggests otherwise.^18^, ^25^ Participants asked candidly, in a conversation they can shape, often consent more durably and recruit others more effectively than participants asked through a standard consent form. The honest ask is not a tax on the public domain. It is the practice that makes a public domain at translation‐relevant scale possible.

The size of the public domain commons and the quality of the conversation that produces each contribution to it are not separate questions; they are the same question seen from two ends. As argued elsewhere, the consent apparatus inherited from the discovery era was not designed with contemporary translational uses in view,^9^ and everything downstream of the public domain depends on the encounter that apparatus governs.

## CONCLUSION

The translational bioethics literature has made substantial progress over the last three years in naming what the field must address: privacy at the level of the biobank record, community standing at the level of the research population, democratic legitimacy at the level of the research agenda, and equitable data sharing across asymmetric research capacities. What remains undertheorized is the piece beneath all of these, which is the substrate of public domain genomic data on which the rest of translational medicine's downstream tools are built.

Choices about that substrate determine which questions are easy to ask and which are hard. They determine which populations are legible to future tools and which are invisible to them. They determine whether the public domain commons grows or is allowed to wither in relative terms as private corporations expand around it. These are ethics questions before they are funding questions. Treating them primarily as funding questions has produced the present situation, in which the translational pipeline depends on a public domain corpus that was adequate for a different era and is not adequate for this one.

The argument of this essay is not that a single project can close the gap. It is that the gap is a proper object of translational bioethics attention, and that closing it requires different commitments from those that built the discovery‐era corpus. Public domain genomic resources are infrastructure of an unusual kind, infrastructure made from people, and the ethical apparatus appropriate to that kind has not yet been built. We need honest enrollment conversations that match the scope of the public domain ask, consent language that names downstream translational uses rather than gesturing at indefinite future research, public domain release that resists enclosure, capacity built inside the communities whose data the resource depends on, and stewardship distributed across the jurisdictions that share the resource's benefits. None of these is a technical fix held in the abstract. Each is a bioethics commitment that produces technical consequences, and the column's readers are among the few people positioned to argue that the field should make those commitments deliberately rather than continuing to inherit them by default.

## ACKNOWLEDGMENTS

The author would like to acknowledge helpful comments from Mark Rothstein, Light Auriga, Chase Yakaboski, and Rebecca Yarvitz. The author also thanks participants in the Bermuda Principles 30th Anniversary Meeting in Hamilton, Bermuda (February 2026) and the Human Genome Organization's Committee on Ethics Law and Society's bioethics workshop in Athens, Greece (April 2026). Importantly, these ideas were honed at the Technical University of Vienna (TU Wien) at a symposium on disruption to research cohosted by the University of California, Irvine, the Austrian Council for Science, Technology, and Innovation, and TU Wien in Vienna in May of 2026.

This work was supported by the National Center for Advancing Translational Sciences’ Clinical and Translational Science Award UM1TR004927 to Eric Vilain and Dan Cooper, and with new faculty setup funds provided by the UC Provost and the UCI School of Medicine's Office of the Dean and Department of Pediatrics, Division of Genetics.
